# Addressing Internalized Weight Bias and Changing Damaged Social Identities for People Living With Obesity

**DOI:** 10.3389/fpsyg.2019.01409

**Published:** 2019-06-26

**Authors:** Ximena Ramos Salas, Mary Forhan, Timothy Caulfield, Arya M. Sharma, Kim D. Raine

**Affiliations:** ^1^Obesity Canada, University of Alberta, Edmonton, AB, Canada; ^2^Faculty of Rehabilitation Medicine, University of Alberta, Edmonton, AB, Canada; ^3^Faculty of Law and School of Public Health, University of Alberta, Edmonton, AB, Canada; ^4^Faculty of Medicine & Dentistry, University of Alberta, Edmonton, AB, Canada

**Keywords:** weight bias, obesity stigma, internalized weight bias, narrative inquiry, narratitive repair model, counterstories

## Abstract

Obesity is a stigmatized disease due to pervasive personal, professional, institutional, and cultural weight bias. Individuals with obesity experience weight bias across their lifespan and settings, which can affect their life chances and significantly impact health and social outcomes. The objectives of this study were to: (a) explore weight bias and stigma experiences of people living with obesity; (b) develop counterstories that can reduce weight bias and stigma; and (c) reflect on current obesity master narratives and identify opportunities for personal, professional, and social change.

**Methods:** Using purposive sampling, we lived alongside and engaged persons with obesity (*n* = 10) in a narrative inquiry on weight bias and obesity stigma. We co-developed interim narrative accounts while applying the three-dimensional narrative inquiry space: (a) temporality; (b) sociality; and (c) place, to find meaning in participants’ experiences. We also applied the narrative repair model to co-create counterstories to resist oppressive master narratives for participants and for people living with obesity in general.

**Results:** We present 10 counterstories, which provide a window into the personal, familial, professional, and social contexts in which weight bias and obesity stigma take place.

**Discussion:** A fundamental driver of participants’ experiences with weight bias is a lack of understanding of obesity, which can lead to internalized weight bias and stigma. Weight bias internalization impacted participants’ emotional responses and triggered feelings of shame, blame, vulnerability, stress, depression, and even suicidal thoughts and acts. Participants’ stories revealed behavioral responses such as avoidance of health promoting behaviors and social isolation. Weight bias internalization also hindered participants’ obesity management process as well as their rehabilitation and recovery strategies. Participants embraced recovery from internalized weight bias by developing self-compassion and self-acceptance and by actively engaging in efforts to resist damaged social identities and demanding respect, dignity, and fair treatment.

**Conclusion:** Narrative inquiry combined with the narrative repair model can be a transformative way to address internalized weight bias and to resist damaged social identities for people living with obesity. By examining experiences, beliefs, values, practices, and relationships that contribute to dominant obesity narratives, we can begin to address some of the socially and institutionally generated negative views of individuals with obesity.

## Introduction

“*We are never more (and sometimes less) than the co-authors of our own narratives…we enter upon a stage and we find ourselves part of an action that was not of our own making*.” *(Alasdair MacIntyre in Linderman-Nelson, p*. *55)*. *(Linderman-Nelson, 2001)*

Weight bias is defined as negative attitudes toward and beliefs about others because of their weight (Puhl et al., 2008). These negative attitudes are manifested by stereotypes and/or prejudice toward people with obesity. Ultimately, weight bias can lead to obesity stigma, which is the social sign or label affixed to an individual who is the victim of prejudice (Browne, 2012). Individuals with obesity experience *external stigma*, which can affect their life chances and significantly impact their health and social outcomes (Phelan et al., 2014). External obesity stigma can lead to devalued social identity that increases vulnerability to loss of status, unfair treatment, discrimination and health and social inequalities (Browne, 2012). Experiencing stigma can impact health promoting behaviors such as avoidance of preventive care, which is counterproductive to public health efforts (Puhl and Heuer, 2010). Weight bias and stigma can also increase both morbidity and mortality (Sutin et al., 2015). Self-stigma or *internalized stigma* can also have adverse health outcomes including poorer health related quality of life (HRQoL) (Latner et al., 2014). Holding negative beliefs about oneself because of one’s weight or size can have a distinct and direct effect on health outcomes, independent of any obesity-related health impairments (Pearl and Puhl, 2016). Weight bias internalization may also mediate poor mental health scores in persons living with obesity (Pearl et al., 2014).

Despite significant research indicating that obesity stigma significantly affects population health outcomes, it has not been recognized as a key determinant of health (Hatzenbuehler et al., 2013; Alberga et al., 2016b; Link and Hatzenbuehler, 2016; Ramos Salas et al., 2017a). This is surprising considering that obesity itself is a global priority and that public health policies to prevent and manage obesity have been established worldwide (WHO, 2000; PHAC, 2011). There are precedents in public health practice for addressing stigma associated with chronic diseases such as mental illness, HIV/AIDS and diabetes (Ramos Salas et al., 2017a). However, there have been very few efforts, either in the public or health domains, to reduce obesity stigma.

There is a general lack of consistency in theoretical frameworks, methodologies and approaches to reduce weight bias and obesity stigma (Daníelsdóttir et al., 2010; Alberga et al., 2016a). To date, weight bias interventions have been primarily focused on reducing external stigma (i.e., changing individuals’ attitudes and beliefs about obesity) and show mixed results. For example, interventions that increase health professionals’ understanding and knowledge about the complex causes of obesity can translate into less blaming of the individual. Such interventions, however, have not been evaluated for long-term sustainability, nor have they been assessed for impact on health professionals’ practices and behaviors (Teachman et al., 2003). Similarly, few interventions to address internalized weight stigma have been implemented and evaluated (Levin et al., 2018; Pearl et al., 2018).

Through a recent critical review of Canadian public health obesity prevention policies and strategies, we showed that current public health narratives may contribute to weight bias and obesity stigma (Ramos Salas et al., 2017b). Specifically, we found that public health obesity prevention narratives, which focus mainly on individual-based behaviors, can simplify the causes of obesity as unhealthy eating and lack of physical activity and contribute to the belief that obesity can be controlled through lifestyle changes. This narrative can cast shame and blame for individuals living with obesity, because it positions obesity as a lifestyle choice. These findings are consistent with other studies from Canada, the United States, and Australia, indicating that individuals with obesity perceive current obesity public health initiatives as overly simplistic, disempowering and stigmatizing (Puhl et al., 2013; Kirk et al., 2014). Considering that weight bias internalization occurs in the context of experiencing stigma through external sources including media, family, school, work, and institutional structures and systems, changing public health narratives may be one way to address both external and internal stigma.

Since the prevalence of weight bias and obesity stigma continues to increase (Andreyeva et al., 2008) there is an urgent need to develop theory-driven interventions (Alberga et al., 2016b). There are a variety of theoretical models that can support weight bias and obesity stigma reduction interventions (Puhl and Heuer, 2010; Hatzenbuehler et al., 2013; Bombak, 2014; Lee et al., 2014; Puhl et al., 2015a,b). However, to date very few theoretical models have involved persons who have experienced weight bias and obesity stigma (Ramos Salas et al., 2017a). One potential model that could be used to involve persons affected by weight bias and obesity stigma was developed by Linderman-Nelson (2001). The *narrative repair model* stipulates that persons who are affected by stigma can be active agents in changing damaged or stigmatized narratives by creating counterstories (Linderman-Nelson, 2001). The premise behind the narrative repair model is that social narratives can shape how we think and how we act (Linderman-Nelson, 2001). In other words, social narratives can influence how we identify groups and populations (i.e., social identity) and how individuals act (i.e., individual agency). Social narratives can create damaged identities for certain groups or populations, which can influence how individuals see themselves, how they act and how they are treated in society.

Social narratives that label human differences can result in stereotypes and prejudice, which can drive stigma (Pescosolido et al., 2008; Thompson and Kumar, 2011). These labels reflect dominant cultural beliefs and create degrees of separation between groups. Labeled persons can in turn experience status loss and discrimination that leads to inequalities through reduced access to social, economic and political power (Teachman and Brownell, 2001). Through the power of counterstories, Linderman-Nelson argues that individuals can resist and replace damaged social identities that have been created about them and for them (Linderman-Nelson, 2001). Specifically, a counterstory “is a story that resists an oppressive identify and attempts to replace it with one that commands respect” (Linderman-Nelson, 2001).

Linderman-Nelson’s model makes it possible to address the effects of both external and internal stigma. For instance, through the process of telling their own stories of weight bias and obesity stigma, individuals may restore their identity and reframe their lives to create a healthier self (Linderman-Nelson, 2001). In addition, others who read their stories and who may have had similar experiences may find it transformative. Finally, by disseminating counterstories among a broader audience, we may be able to create social and political messages about the way that society defines and treats people with obesity. Thus, counteracting master narratives about obesity and about people with obesity may transform the way we all think about obesity.

## Objectives

The objectives of this study were to:

(a) Explore weight bias and obesity stigma experiences of people living with obesity;
(b) Develop person-centered counterstories to reduce weight bias and obesity stigma; and
(c) Reflect on opportunities for personal, professional practice and social change.

## Materials and Methods

### Narrative Inquiry Methodology

Using narrative inquiry (Clandinin and Connelly, 2000), we engaged participants in conversations about their experiences (stories) with obesity, weight bias and stigma, focusing on personal, public, and health care domains. The goal of narrative inquiry is to allow participants to find meaning in their own experiences (as well as those of many others in similar situations) by telling and retelling their stories over time. The premise of narrative inquiry is that people live storied lives and that by investigating into our experiences (stories), we create a new vantage point from which we can understand and learn from our own experiences and those of others. Together, participants and researchers, may create coherence between the stories and also find meaning within the stories (Clandinin, 2013).

The process of narrative inquiry is inherently relational and collaborative. As participants and researchers lived alongside each other, we (including the lead researcher) shared our experiences with obesity and weight bias across times and settings. The lead researcher kept a journal and recorded field notes. Some participants provided other data sources such as pictures and other memory artifacts that could help us develop in-depth narrative accounts. Each participant met with the lead researcher several times.

After developing *interim research texts* in collaboration with participants, the lead researcher invited participants to provide feedback and to share their personal responses to all the stories. We asked participants to be attentive to their own emotions and to consider the potential silences in the stories, which may also reveal critical meanings. Together, participants and the lead researcher, read and re-read the stories to uncover personal, familial, social and institutional contexts that have shaped our shared experiences of obesity, weight bias and stigma.

We used the *three-dimensional narrative inquiry space* to organize each story according to: (a) *temporality* (i.e., stories are always in transition and are linked through the past, present and future), (b) *sociality* (i.e., stories are informed by the personal and social conditions in which we live) and (c) *place* (i.e., stories happen within physical and topological boundaries where the inquiry and events take place) (Clandinin, 2013). The three-dimensional narrative inquiry space provided a conceptual framework that helped us understand how obesity and weight bias experiences (stories) happen in the context of time, social milieu, and physical space. This framework also allowed us to create coherence between the stories and find meaning within each story. Ultimately, this collaborative analysis and interpretation process helped us realized that some situations were not unique and that many people living with obesity go through similar experiences of weight bias (including internalized weight bias), stigmatization, and discrimination. After identifying coherences and meanings, the lead researcher developed the *final research texts* with full participation from all research participants.

**FIGURE 1:**
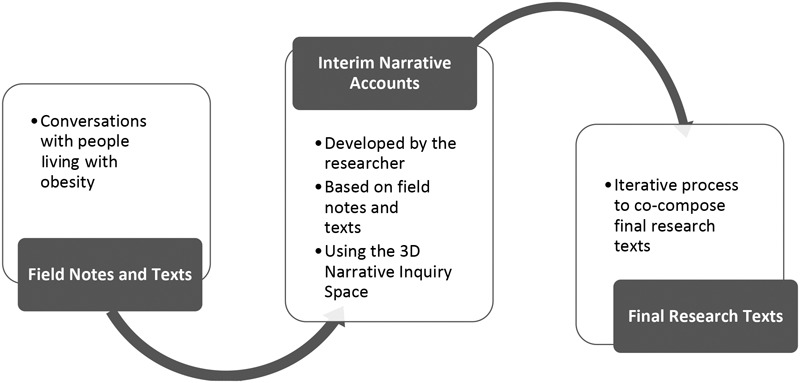
Narrative inquiry process.

This narrative inquiry process took place over 2 years (Figure 1). The prolonged and intensive engagement with participants was central to the research process, as it allowed the lead researcher to interact with participants and determine the degree to which they were comfortable with the final research texts and to gauge how participants’ perspectives shifted through the research process.

In this paper, we share counterstories (final research texts) developed by 10 individuals living with obesity who have experienced weight bias, stigma, and discrimination. We invite the reader to read these stories and to place themselves within them, reflecting on their own stories as well as our collective master narratives of obesity. Examining experiences, beliefs, values, practices and relationships that contribute to dominant obesity narratives has been recommended as a way to address some of the social and institutionally generated negative views of individuals with obesity (Aston et al., 2012). Reflexivity may also help challenge social narratives about obesity and change personal attitudes, beliefs and practices about obesity prevention and management (Aston et al., 2012).

### Participants

Using purposive sampling, we engaged persons living with obesity (*n* = 10) from various Canadian provinces in a narrative inquiry to construct and interpret participants’ experiences with obesity prevention policies, weight bias and obesity stigma. Since, the purpose of this study was to explore weight bias and stigma experiences of people living with obesity, we used the Obesity Canada’s definition of obesity (OC, 2013) to recruit participants. Obesity Canada defines obesity as a chronic disease characterized by excess or abnormal body fat that impairs health, regardless of body size. All participants in this study self-identified as having obesity and had also been diagnosed with obesity by a qualified health professional. No anthropometric measures were necessary because all participants had clinical obesity.

All participants were over 18 years of age. Participation was voluntary, and participants were recruited through bariatric clinics and through Obesity Canada (formerly the Canadian Obesity Network) – a national registered charity with a mandate to translate research into policy and practice. Participants did not receive financial compensation for their participation in this study. Each participant was assigned a pseudonym.

### Ethics Statement

We obtained ethics approval from the University of Alberta Health Research Ethics Board. All participants received a study information package prior to the conversations. All participants also provided written informed consent for the purposes of research participation as well as for the publication of the counterstories. Participants were informed that pseudonyms would be used in the stories.

## Results

### Catherine: Counteracting My Unhealthy Social Identity

My doctor told me that obesity is a chronic disease, meaning that I will live with obesity for the rest of my life. Right now, he says I am in remission because I am managing my disease well enough that my weight does not impact my health. Even though I am healthy and at my best weight, I am still actively managing my weight because if I stop the treatment, the weight will come back.

However, my friends and family still think I should lose more weight and still make comments about my “unhealthy weight.” These unwanted comments never stop. People are well meaning but they have no idea what I am going through. They just see my body and assume that I am unhealthy. I cannot change the size of my body. This is the body I have learned to accept after years of abusing it and trying to make it “*normal*.”

I believe the messages about “*healthy weight*” have certainly contributed to my weight bias experiences. Society tells us that obesity is bad and that people with obesity are a burden to society. This narrative has an impact on social attitudes, which has had a direct impact on me as an individual. Wherever I go, the first impression I make in someone is that I am unhealthy. This allows people to treat me differently. When I walk down the street, strangers tell me: “You should not be eating that” or “You should take the stairs instead of the elevator.” My body is identified by others as “*unhealthy*” and over time, I started to believe that my body was unhealthy and that I was unhealthy.

Everything I have done to lose weight is because I wanted to become healthy. As an indoor cycling instructor, for example, I am always looking for new places to work. During a job interview few years ago, an employer asked me if I considered myself to be healthy. I was taken aback with that question and I asked her to clarify what she meant. She asked: “Do you think your weight is healthy”? This is how our society views me. So, I need to make sure that I change that perception by sharing some of my journey with them.

I tell them that I eat healthy based on what my bariatric dietitian has recommended and that I exercise as much as I can. I teach 4–6 cycling classes per week and run in as many races for which I have time in my busy life as a mother, wife and teacher. I take medication to manage my obesity just like people with diabetes take medications to manage blood sugar levels. I see a bariatric doctor regularly and I although I still live in a large body, I am healthy.

I also tell them about my journey in accepting my body and treating myself with respect. I share some of my experiences with weight bias and bullying. Every weight bias experience is like a mini-trauma. It leaves a mark and makes it is harder to recover from them. But I have developed a “thick skin”. These mini-traumatic experiences have become part of my life. They have made me stronger. But that does not make it right. Looking back on those experiences of weight bias, bullying, teasing and abuse, I can tell that the stigma scars are permanently imprinted in my brain. Sometimes, I need to put a lot of effort to prevent these scars from affecting my life. Negative self-talk and poor body confidence are barriers to my emotional health and my disease management process.

Another way I use to fight weight bias is to explain to people that shaming people for their body size is not helpful. It has never helped me to change my behavior. The more I feel badly about myself and my body, the more I avoid healthy living activities such as going to the gym. Through this story, I want to let people know that making someone feel bad about themselves is detrimental. We should encourage and support others who feel unwelcomed in fitness facilities, for example, because after all, exercise is good for everyone – not just for people with obesity.

### Caroline: Obesity Is Not a Lifestyle Choice!

In grade two or three, I missed a vaccination day in school and my mother had to take me to the public health clinic instead. The nurse weighted me in the waiting room in front of everyone and told my mother that I was obese. I will never forget this experience. Once we were in a private room, the nurse scolded my mother because I was obese. My mother asked the nurse what she should do. The nurse told her that she needed to put me on a healthy diet and exercise program. That was the beginning of my weight loss career. I call it a career because I truly have been working at my weight all my life. But no matter what I tried, the weight always came back.

As an adult, I finally found a doctor that specialized in obesity. She took the time to explain to me what obesity is, and I realized that a lot of what I knew about weight and health was incorrect. I was obsessed with losing weight through diet and exercise because I wanted to be “healthy.” In retrospect, I just wanted to be normal. But after all those years of yo–yo dieting and exercising, I had actually damaged both my soul and body. I had convinced myself that there was something wrong with me because I could not manage my weight.

Once I realized that I had a chronic disease, I took every opportunity to learn more about it. I even went to an obesity conference to inform myself about the latest science on obesity. This is when I realized how much damage I had done to my body, my metabolism and my soul.

One of the most important things I have learned is that obesity is a complex chronic disease and not a lifestyle choice. The Canadian Medical Association (since 2015), the American Medical Association (since 2013), the World Health Organization (since 1948), Obesity Canada (formerly known as the Canadian Obesity Network) (since 2011), The Obesity Society (since 2013), the Obesity Action Coalition, the European Association for the Study of Obesity (since 2013) and the World Obesity Federation (since 2017) are just a few organizations that have recognized that obesity is a chronic disease. For years, I have been told by health professionals, friends, family, and the media that obesity is about my lifestyle (unhealthy eating and lack of exercise). Well, I have tried healthy eating and exercise plans all my life and that has not changed my obesity. For 20 years, I tried that. Yet, I still have obesity.

For my mother, having a daughter that had been labeled as obese was devastating. She tried to make me healthy in her own way. She cooked different foods for me. While everyone else was eating regular meals, I was eating soup or raw vegetables. Whatever her friends told her to try, she tried it on me. I resented the fact that everyone could eat whatever they wanted, while I was stuck on an endless diet. My mother put me in dance classes, swimming, power skating, and even made me do exercises at home such as running up and down the stairs or running around the backyard.

When I deconstruct the current narrative about obesity, it says to me that health professionals blamed my mother and me and so they did not need to help me find evidence-based treatments. My mother internalized this message and truly believed it was her fault that I had become obese and that it was her responsibility to fix it. I also internalized the weight loss failures and truly believed that there was something wrong with me.

It took years for me to realize that there is nothing wrong with me as a person. That my identity is not defined by my weight. That I am a human being who deserves respect and dignity. That I have a chronic disease that requires long-term management, and that it is my right to expect respectful and dignified care.

Today I am making it my mission in life to prevent weight bias and obesity stigma. People need to understand that obesity is not a lifestyle choice. I did not choose to have obesity. In fact, I suspect that my obesity can be linked back to years of yo–yo dieting as a child, which have reduced my metabolism (maybe permanently) and made my body much more energy efficient. Years and years of losing and gaining weight may have also increased my weight set point, which makes my body counteract any weight management by releasing a cascade of hormones designed to protect against weight loss.

Health professionals have a responsibility to educate the public about obesity in a holistic way. Obesity is not just about the food environment and lifestyle changes. We now know that weight is also intrinsically linked to genetic and biological mechanisms. Rather than presenting obesity solutions as “eat less and move more” strategies, health professionals should adopt the consensus of the medical community and international health agencies that obesity is a complex chronic disease that requires prevention and management strategies that are evidence-based. Simply telling people to “eat less and move more” is frankly unethical, considering that yo–yo dieting and exercising can cause major damage to individuals’ mental and physical health.

### Sarah: Resisting Institutionalized Stigma and Changing the Way We Accommodate People With Obesity – A Story Narrated From the Perspective of a Weight Bias Researcher!

Sarah exudes confidence and happiness as she comes into my office. I welcome her with a hug but the atmosphere changes quickly when I realize my office does not have chairs to accommodate her body. Sarah laughs it off and tells me:

“*I am used to not finding seating*. *The first thing I do when I walk into a room is to scan for chairs*. *If I do not find one that fits my body, I prefer to stand*. *The looks I get when I try to fit in a chair are just not worth it*.”

I am shocked that this state-of-the-art obesity research institution lacks seats for people with obesity. The next time we meet, it is at a coffee shop with more comfortable seating. But, there I notice people turning to look at us. Their critical faces show judgment.

“*I am used to those looks too*. *These looks can become verbal attacks sometimes*. *Strangers will stop me on the street and tell me that I should eat less and exercise more*.”

I am uncomfortable and want to leave. I want to protect her from this experience, but I realize that this is part of our story. While living along Sarah, I am traveling into her world. Weight bias and stigma are common experiences that have shaped her life. She tells me about her dream to become a teacher and how her dream almost fell apart on the first day of university. As she entered the classroom she realized the chairs had built-in tables around them and that her body would not fit. Before people could notice she was in the room, she left the classroom. She felt like never returning, but her dream of becoming a teacher motivated her to find a solution.

The next day, she placed a regular armless chair in the back of the room. This became one of her safe place on campus. Over time, she found a few more places where she could sit and study. These spaces represented her own resistance to physical barriers that limit her participation in life. As she shares this personal story with me, she knows that she is creating a resistance against the social exclusion of larger bodies.

We discuss how bodies are marginalized in our society and how people of size are affected.

“*The message is that I do not belong here because I have obesity*. *The stories tell the world that we are unhealthy, lazy, unmotivated, unintelligent, disgusting and ugly*. *People make judgements about my moral character because of my size*. *The assumption is that I did this to myself*. *It is my fault*. *I somehow lack the discipline and willpower to be healthy*. *That I am a burden to society or that I do not contribute to society*. *This gives people the right to exclude me from participating in society to my full potential*. *But everything I have done to my body is to become healthy*. *I had bariatric surgery because I wanted to become healthy*. *But that is not enough because I will never be considered a* “*healthy weight*.”

We explore the idea of healthy weight a bit further. As she tells me her experiences with fat shaming from family, friends, colleagues and strangers, she reflects on how she has internalized these harmful stereotypes and attitudes. Unconsciously, she internalized a harmful personal identity which has shaped her life. Despite having lost a significant amount of weight after her surgery, her body is still classified as obese. However, her bariatric physician recommended that she not lose more weight because it could have negative consequences for her overall health.

“*He told me that I have reached my best weight*. *He defines best weight as the weight at which I can be healthy and live happily*. *And I agree with him*. *But my family doctor keeps telling me to continue to lose weight – to eat less and move more*. *But how am I supposed to do that when I am already eating the least amount of food that my body needs to function? How am I supposed to fit in more time for physical activity when I am already exercising 2 h each day? Clearly, my family doctor expects me to reach a certain number on the scale as if that number will make me healthier*.”

Sarah believes that the idea of “*healthy weight*” comes from public health messages which are contributing to the internalization of weight bias among people with obesity.

“*What does* “*healthy weight*” *mean? I am healthy right now but when people look at me they assume that I am not*. *I don’t focus on the number on the scale anymore*. *I count my non-scale victories*. *I focus on the activities that I can do now which I could not do before, like going on a roller coaster or going on a vacation or getting a new job*. *I also work hard to be as healthy as I can be by exercising and eating healthy foods*.”

Sarah has seen what internalized weight bias can do to someone. Unhealthy weight loss practices are common. She tells me about a friend that goes to the gym three times per day (before work, at lunch time, and after work).

“*She became obsessed with her weight loss and alienated everyone in her life in the process*. *I do not want to do that*.”

We discuss this further and question why people with obesity are held to a different moral standard than others who have chronic diseases such as cancer, heart disease or hypertension.

“*Weight is supposed to be under my control*. *I have the control to change my body weight because I choose what I eat and how much I exercise*. *That’s what most people believe*. *I believed that too*. *It was not until I started learning about obesity that I realized that there are many factors that influence my weight and the choices I have*. *I used to blame myself every time I gained weight*. *But I did not know that lack of sleep was affecting my hormones, for example*. *I did not know that the medication I was taking for depression was making me gain weight*. *There is such little awareness about obesity in our society*.”

The assumptions we make about people’s moral character are based on lack of knowledge about obesity. We discuss this further as we sit in this now crowded coffee shop. The smell of coffee and pastries is filling our senses and we are enjoying each other’s company. We are connecting as humans and we are sharing an experience that is filled with empathy and respect. I notice that a family with two young children sits next to us. They are staring at us and whispering to each other. They look at our plates and I notice they are making comments about our lemon pie. I noticed that both the parents and the children keep looking at Sarah’s body as she repositions on the couch. Sarah is also aware of the looks. I make a comment about the lemon pie, desperately trying to stop thinking about this family who is staring at us. Sarah senses my discomfort and says:

“*I am aware of the looks*. *It happens in almost every restaurant*. *As if I somehow do not have the right to eat pie because I have obesity*. *Mostly everyone in this coffee shop is eating some kind of pastry with their coffee, but do they feel judged as I do, I wonder? Is this in my head? Am I imagining the stares?*”.

No, she is not imagining the judgmental looks. I can see them too. In fact, if I am honest, I tell her, I may have done this myself. In my training as a kinesiologist, I learned all about the “energy in and energy out” model. That is as much as I learned about obesity in my 4 years of undergraduate studies. During my Masters’ degree, there was no learning about obesity as a chronic disease. Instead, obesity was seen as a risk factor for other chronic diseases. So, the lack of understanding of obesity in the general public is similar to the lack of training and awareness among health professionals. This is why I am doing this research study, I tell her.

“*I am thankful to you for sharing this experience with me*. *I can see how it has changed both of us*. *I wish other researchers would engage people living with obesity in their work to create more change in our society*.”

### Louise: Weight Bias and Obesity Stigma – It’s About Life and Death!

I had a nice childhood. A nice family. I have a successful career. I am self-disciplined. But, obesity runs in my family. My parents and grandparents had it. However, my sister does not. She can eat whatever she wants and never exercises. I guess I got the obesity genes in the family. I am managing my obesity well. I was lucky to have a good primary care team that helped me. But I still have a high BMI and I feel like there is something wrong with me and that I need to lose more weight. Somehow my value as a human being is lower than someone whose weight is considered “normal.”

Thinking back, I realize that my parents have been telling me that I need to lose weight since I was a child. Even though I ate healthy foods and I participated in extracurricular activities such as swimming and running, they told me that I need to eat healthier and exercise more. As a teenager, my parents questioned my eating habits and accused me of hiding junk food in my room. I never hid food in my room. But, I remember going to bed hungry because I was not eating enough before and after my swimming or running practices. It hurt that my parents did not believe me, but they did not know better. If my parents had known what we know today about the biology of weight gain, they would not have done that because they love me. I know that. There is no doubt that I have scars from my childhood. My relationship with my parents was damaged. To this day, they comment on my weight, my eating habits and my appearance.

Public health messages about obesity make weight control sound easy. But, my journey has not been easy. Every day is difficult. I have lost over 100 lbs. and I am managing my disease well, but I still have obesity according to the BMI categories. My goal is to maintain this weight loss. Based on what I am doing now, I cannot eat less and I cannot exercise more. So, I will never achieve the healthy weight range promoted through public health campaigns. I am at my “best weight” and I need to accept that. Why is public health not OK with that?

The idea that we need to pursue a “*healthy weight*” or a “*healthy BMI*” is not relevant to most people living with obesity. There is a lack of recognition in public health that people come in different shapes and sizes. In my view, public health obesity prevention strategies should not emphasize body weight or BMI (size). Public health should aim to improve health. Size and BMI are not health outcomes.

Public health efforts should aim to support people with obesity, but we are not part of the public health policy making process. We are often excluded from health policies. Obesity prevention strategies target individuals who are “normal” weight in order to prevent them from becoming “obese”. But what about those living with obesity already? Who will help us?

Finding obesity care in Canada is challenging, to say the least. Most health care professionals have not been trained in obesity and will simply advise their patients to “eat less and move more.” So, we are left to fix this ourselves. How is this acceptable? Well, it is acceptable because people with obesity are not valued in society. We are seen as lazy, stupid and dishonest individuals that simply cannot adopt public health messages and strategies. Obesity is our fault. Those are the assumptions that people make about us. Well, it is time for us to change those assumptions. People with obesity deserve to be treated with respect, just like everyone else. What matters is that we need support.

The way health professionals think about obesity has a direct impact on my health and well-being. A direct example of this in my case was when my doctor blamed the back pain I was experiencing on my obesity. He dismissed my complaints and I lived with pain for over 2 years, until I decided to get a second opinion. I went to see another doctor who has been trained in obesity and he completed a full health assessment, without any moral judgment or preconceived ideas about my weight. After a few weeks of medical tests, we discovered that I had kidney cancer. By the time we discovered the cancer, it had progressed to stage 2. I was angry and upset. The cancer could have been discovered earlier if it was not because my previous doctor believed that I just needed to lose weight and my back pain would go away.

Never mind finding adequate evidence-based obesity treatment and care within the current Canadian health care system. We can’t even find dignified health care in general. Every time a medical problem is blamed on obesity, we experience bias and discrimination. This can have serious health consequences for us as individuals.

The majority of the time, health care professionals do not make assumptions about how someone developed diabetes or cancer or heart disease. But they assume that individuals with obesity are eating too much and exercising too little. This thinking leads to disrespectful treatment and poor quality of care. People with obesity need to challenge these assumptions and share their stories. We need to change the social identity that has been created for us and regain our moral value as human beings.

### Karen: Does My Life Matter to Public Health Decision Makers?

My grandfather used to bribe me so I would stop eating. He used to give me money if I skipped dinner. He would say – “See? You just need motivation and you can lose weight.” But I was starving, and my body became extremely efficient at storing fat. The more I restricted my eating, the more weight I gained. Diets never worked. My parents started telling me that I needed to take responsibility for my own decisions and that they would no longer help me. I never felt supported by my family. Today, I am still hurting because my family believed that I did this to myself.

In school, kids called me names and abused me physically and mentally. One day in grade 6, a boy in my class walked up to me and spit in my face and yelled “you disgust me, why don’t you lose some weight?” I was in shock and could not say anything, so I turned around to walk away but he pulled my hair and I fell to the floor. He then proceeded to kick me in the stomach while continuing to yell at me “you are a disgusting pig.” Nobody did anything. There were other kids watching the whole thing, laughing I was crying and yelling at him to please stop but he kept kicking me. He eventually stopped and walked away but not before spitting in my face one last time. I started skipping school and avoided being alone around school. I had no friends and so often I hid in the bathroom during recess so that kids would not see me. This abuse went on for years. There were times when I wanted to die. I stopped telling my mother about the bullying in school because she would just put me on another diet. That was her way of trying to help me.

After years of abuse and isolation, I began to comfort myself with food. The weight gain continued. I suffered in silence and I was relieved when my mother’s new boyfriend, who also had obesity, joined our family and we could have conversations about our shared experiences with bullying. I trusted him. The first time he raped me, he threatened to tell my mother that I was skipping school. The threats became worse every time he raped me. I was broken. I was alone. I had no one to trust.

By the time I was 15, I had lost the will to live. I ran away from home and had nowhere to go. Soon, the darkness of the streets consumed my life. Drugs, sexual violence, and crime became part of my life. When I was 17, I was raped, beaten, and left for dead on the streets. A public health nurse found me and took me to a safe place. She saved my life. Today, I am working to address homelessness in my community. I found my voice and I want to give a voice to others.

Obesity has been part of my life and I continue to struggle to manage my weight. But the isolation, abuse and violence that I experienced has changed me. As an isolated, lonely child with obesity, I was more vulnerable to sexual predators. We need to protect our children from adverse childhood experiences. We need to help them before it gets out of control. I hope that my personal experience living with obesity and experiencing shame, blame and abuse can help others. There is no question in my mind that obesity stigma can lead to experiences of social exclusion, abuse and discrimination that ultimately leads to health and social inequalities.

Public health could have taken away the pop and junk food from my school cafeteria. They could have influenced the food environment in my community. I am sure that would help many people. But, I would have still gone out to buy these items from the local convenience store. Yes, I ate unhealthy foods throughout my childhood. It was how I coped with the abuse. I did not choose to experience physical and sexual abuse as a child. But I chose to eat junk foods. *It was all I felt I had control over*. So, yes, I guess obesity is my fault. I did this to myself. But does it matter? Does that give people the right to treat me without respect? Does that mean that public health strategies do not need to take me into account? Is it too late for me? Does my life not matter?

I hope my journey helps health professionals understand that there are many causes of obesity. Obesity prevention strategies should address the true causes of obesity. In my case, the underlying factor for my obesity was shame, trauma and abuse. These are psychological factors that should have been addressed early on. Prevention in my journey should have involved psychosocial support – not just diets and exercise programs.

The bottom line is that health professionals need to understand that a healthy lifestyle is just one component of obesity. So, the question is: what is public health doing to specifically prevent obesity (other than promoting healthy lifestyles)? How is public health addressing the many underlying psychosocial causes of obesity? How is it addressing the realities that people with obesity experience?

### Steve: Finding a Community and Changing Obesity Narratives

I may not have been here today if I had not had bariatric surgery 10 years ago. When I think back at the 3 months I spent in the hospital because of complications, I realize that I was put in this world for a specific reason.

My obesity journey did not start with surgery. My journey started so many years ago when I was a young boy living with my mother who raised a family the best way she could. She showed loved with food, and I needed that love so much. I don’t think she ever knew how much I needed her love. A mother is the person who is always there for you. But my mother was not able to be there for me due to her experiences with depression. Reflecting on my childhood I realize that so much of my journey started with my mother.

I don’t have resentment toward her now. But, for a long time during my teenage years and early adulthood, I resented her for not protecting me from my abuser who inflicted so much pain on me. I was the target of sexual abuse for years. The only thing I could find comfort in was food. I used it as comfort, as love, as a mechanism to change my body and become invisible. The larger my body became, the more invisible I was to the world – I hoped.

I have been ashamed to speak of this to anyone. Until now. I am turning the page. I am free from this past. I am looking to the future where I can share my story to make a difference in this world. Looking back, I can see how my experiences have created a scar in me, like the one from the bariatric surgery. The scars are not just physical. The scars are also emotional. They will always be there to remind me of where I have been and how far I have come.

Reflecting on my childhood, I can see that all I wanted to do was to run away from that world. I tried to erase childhood memories and used food to feel in control. I wanted to have control of my life. And yes, doctors warned me that I was “morbidly obese.” I hated hearing those words because it made me sound like a monster. But I am not a monster. I am a human being in search for love and belonging.

I continued to gain weight and along with it came the experiences of bias and stigma. Some would say to me: “*You are being reckless with your body and health*. *Get a hold of yourself*. *Wake up or you will kill yourself*.” Even when I was waiting for bariatric surgery, strangers, health care professionals, family and friends looked at me with disgust and contempt. They judged me and created their own stories about me. Stories about me lacking discipline, being stupid, and not caring about myself. Stories about my food addiction and my inability to control myself. One day, I realized that these stories about me were hurting my health.

For example, I was accused of lying about my food intake countless of times by healthcare professionals. I was blamed for my obesity over and over again by healthcare professionals who believed I was acting recklessly and did not care about my life. I was shamed for my obesity in hospitals when told that I could not get diagnostic tests because my body did not fit in hospital equipment.

This is why I decided to share my story. This is not who I am. I am an intelligent person, I care about myself, and I want to have a healthier life. Finding a community of people living with obesity who have experienced similar stigmatization and discrimination in schools, workplaces, and health care has helped me. I have re-gained my sense of belonging. By telling and retelling my story, I am reliving my story and I can see the places, times, and relationships that shaped my life and my obesity journey.

The story about me choosing to develop obesity because I didn’t care about myself is not true. I did not choose to do this to myself. Nobody chooses to do this to themselves. Obesity is not a choice. Every person with obesity has their own story, which means that each person needs a different type of support. Those of us who can need to share our stories so that we can help our community. People need to hear these stories without judging them and without imposing their own biases on them. We are human beings and we all deserve respect and dignity.

### Nancy: You Cannot Empower Me, I Can Empower Myself!

My experiences with weight bias go deeper than I had ever thought. The stories of weight bias are within me. They are part of me. Years and years of bullying in schools, physically abusive relationships and unfair treatment at work led to feelings of isolation, loneliness, and not belonging. These experiences have changed who I am and have shaped my life.

My earliest experience of weight bias was from my mother. My mother loved me and wanted the best for me. She wanted me to be healthy and marry a good husband, so I could have a beautiful and happy family. The love of a mother is undeniable.

My mother put me on my first diet when I was 10 years old. At the time, I did not think I was chubby or fat. I was a normal little girl who played outdoors all day on our family farm. I ate homemade meals with fresh produce. I played sports in school and loved art classes. I was a curious child and would explore my family farm every day. I loved being outdoors and found ways to create a friendly world. I would stay outdoors so I would not have to hear my mother’s comments about my weight, to be safe and happy in my own world.

Despite seeing doctor after doctor, trying diet after diet, practicing, and performing dance after dance, my weight continued to increase. My mother was worried about my weight and my health. But she was also worried I would not be able to find a husband who would love me because of my weight. This story went on for years and it became my story. I used to think: “*will anyone love me? is there something wrong with me? how can I be such as disappointment to my mother who loves me so much? I just need to try harder*.”

And I did everything I was told. I tried every diet and exercise program she told me to try. I was my mother’s project. She tried and tried to change my weight, so I could be beautiful and healthy. But nothing worked. The weight would come off and then it would come back again. I did it again and again, like a yo–yo. Hundreds of pounds gained and lost throughout my life. My ideal of beauty became about weight. My mother never said I was ugly. She just kept trying to make me more beautiful. Her story became my story. I tried and tried to change my body.

Soon, everyone in my family would tell me to lose weight or nobody would love me or marry me. They would recommend diets, exercise programs and/or doctors. Little by little, I started to believe this story. There is something wrong with me. I cannot lose weight because I am stupid, lazy, and unmotivated. I am not like the rest of the world. I cannot do simple things like eating healthy and exercising long enough to keep the weight off. Everyone else can do it, except me. It is just me. I am alone.

Despite my mother’s fear that I would not meet a boy who loved me, I did. I moved in with him only to experience another form of weight bias. My boyfriend said he loved me and then he stopped buying food. We had no food at home and my weight went down, way down. I became underweight. I was at my lowest weight ever because my boyfriend who loved me would not buy food. I had nowhere to go. I thought this was love. This is what my mother wanted me to find. She wanted me to find love. Taking away the food was just the start. Soon the abuse became physical and emotional. How is this love? Why does love feel so lonely? I eventually I left him. This was not the story I wanted to live. I needed to change my story.

I went back to university to finish my degree and started a career in business. I achieved tremendous success as I put all my energy toward my career. Slowly the weight came back. The stressful career and the stories I had left behind just made the weight come back. My brain re-claimed the weight it had lost and put on even more weight. But this time, the weight started to affect my health. Diabetes, hypertension, sleep apnea, bone and muscle pain coupled with anxiety and depression became part of my story.

By this time, I had re-married and had a daughter of my own. I wanted to be there for her. I tried so hard not to make her story about her weight. I wanted her to be strong and confident. But, in re-writing my story, I lost track of my health. It was time to reclaim my health. This time I realized that years and years of yo–yo dieting, shaming, weight bias, abuse, and loneliness had taken a toll on my body and health. My metabolism was destroyed. My weight was out of control and my health was suffering. I took control and empowered myself to seek support. This is something that health professionals needs to understand: they cannot empower others. Empowerment is internal. It cannot be given to others. All health professionals can do is to provide support and respect. I reached out to a medical expert. I refused to try another diet. This time, I realized that I needed help from someone who understands obesity. It is not about looking skinny or beautiful to me. It is about my health. It is because I need to be healthy and live a long life with my daughter, son, and grandchildren.

But, wait. To see the look in my husband’s face when I undress. That is also important. He is a loving husband and has never said anything about my weight. But, the looks on his face reinforce all the shame and blame I feel. *Could it be that the person that I trust the most shares the worst beliefs I have about myself? Does he believe the same things about me that the rest of world does? Does he also believe that I don’t have self-control and that I did this to myself?*

At work, I tried to implement healthy snacks. But my colleagues tell me that I need to find a way to control my own impulses and that I cannot take it out on them. Just because I need to lose weight does not mean they cannot eat doughnuts. I argued that having healthy meeting snacks is good for everyone, but they don’t see it like that. I did it to myself and it is my fault. They don’t need to eat healthy. It is my problem.

By now, I have reflected on my story. Weight bias was one of the main drivers of my weight gain. I know that my mother’s sense of love for me was expressed through weight bias. I am a human being who deserves to be loved no matter what size I am. I also deserve to have access to the right support to manage and improve my health and to change my future. Today, I am focused on my health and my life. I have a new job that keeps me active and that promotes healthy food environments. I can go to work and trust that people will stop offering me unhealthy foods or any food, for that matter. They respect my journey and my story and want to be supportive. I wish every person living with obesity would have this type of supportive environment, where they can be themselves and where they can be the healthiest and happiest that they can be. Where they are in control of their story!

People with obesity have different stories and cannot be put into one box. We need to listen to those stories and create environments in which every story can flourish. People with obesity want to be healthy, loved and respected just like everyone else. Health professionals cannot just focus on the weight. Obesity prevention and management strategies are needed but they cannot be measured against weight loss or reductions in Body Mass Index.

If health professionals do not change that narrative, people like my mother will continue to believe that to be healthy or loved you need to be skinny. And that people with obesity cannot be healthy or loved. That people with obesity need to change to become “*normal weight*” or “*healthy weight*.”

I am living with a chronic disease that I need to manage every day. Every day, I need to think about my food decisions, my exercise levels, my stress levels, my sleep, and my emotional health. I will never be considered a “*healthy weight*” and I will never have a “*healthy body mass index*” but I have lost the weight that was impairing my health and now I can live my life. I can be there for my children and my grandchildren. I can be loved by husband and live a happy life. I don’t need to be skinny to be healthy and loved.

### Margaret: Shame and Internalized Bias

Shame penetrates every part of your body. It penetrates your mind deeply. Shame triggers deep feelings of inadequacy, guilt, and vulnerability. Shame hides in your mind and you feel out of control. When you feel out of control, you can do a lot of damage to yourself.

Shame also channels into your heart and you stop loving yourself. You start believing that nobody can love you because you don’t love yourself. This leads to loneliness. Shame gets into your gut, triggering the shame triggers or the hunger hormones. All you can do is feed those triggers to calm them down.

But every time you feel shame and lose control, you lose a bit of yourself and you feel yourself changing slowly. But you get up and you go to work, you take the kids to school and you start another diet and exercise program. Each time you fail, the shame increases, until one day your weight and shame affect your health and you get sick: a stroke, a heart attack, diabetes, back pain, knee pain, depression. One day, you realize that you could die from obesity and shame. But where do you go for help?

Society tells you it is easy. You just need to eat less and move more. But, how do I deal with the shame in my mind, my heart and my gut? Nobody is there to help me. I am alone.

And I try again, and again, but nobody can do this alone. It is a basic human need to have someone to trust to rely on for help. But all I hear is: “*you did this to yourself and you need to get your act together and figure it out alone*.” “*Nobody can help you unless you want to help yourself*,” they say. What does that mean? Would you say that to someone who has cancer? Does that work for anyone who has mental health issues? Does it work for anyone out there?

I walk down the street and the stereotypes about obesity are everywhere: in my family, in my school, in my workplace, in my local fitness center. I can never get away from those stereotypes. I even believe these stereotypes. I live those stereotypes.

Weight bias is about shame. I am ashamed of my body. I am ashamed for my failure to control my weight. But the reality is that our brain and gut will work together to counteract weight loss and defend the highest weight at all cost. On one hand, this is a positive scientific finding because it shows that weight regain is not the result of lack of will power, commitment, or effort. Unlike what my friends, family, and bariatric specialists believe, I am not lying about my food intake or physical activity levels. My body is simply very efficient at counteracting my weight loss efforts. That is the bad news about this scientific finding. Significant biological mechanisms will counteract every change I make, making weight loss maintenance even harder.

These compensatory biological mechanisms are not well understood by scientists, but as an individual living with obesity who has tried to lose weight all my life, I can certainly attest to them. Each time I lose weight, I feel hungry and my body temperature goes down. My body becomes way more efficient at storing fat and although I am still running the same distance and eating the same number of calories, my weight loss will either stop or I will start regaining weight. This means that if I want to sustain the weight loss, I need to reduce my calories even more and I need to spend more calories by exercising even more. But, there is a limit to how much I can increase this effort. Any person trying to sustain this effort would struggle. It is not impossible, but it is hard.

When I reflect on the shame that I experience every time I regain weight, I realize that this is unfair. I am not a failure. I simply do not have the right tools and support to manage this chronic, relapsing disease. What if I was living with hypertension? Would I be expected to manage my blood pressure on my own through diet and exercise? No, my doctor would first give me medications and then support me to make behavior changes. But because this is obesity and I should have control of my weight, I am expected to manage obesity on my own. Forget all the compensatory mechanisms working against me. Those are just excuses, and I just need to try harder.

### Andrew: It’s ‘Us’ Versus ‘Them’

I have a room full of trophies and medals that remind me of my hockey career. I remember the early morning and late evening practices, the weekend journeys to hockey tournaments and the many hockey camps I participated in. But, the memory of the day I broke my ankle is more vivid than any other. It was the end of my hockey career. Everything I had was gone from that moment. Although, doctors, family, and friends supported me and gave me hope that I could play again, I knew this was the end. It felt like it was the end of my life. I developed severe depression and became isolated and alone. Doctors put me on antidepressants but they did not really help. By now, I had missed so much of school that I could not finish the school year. I dropped out and hid away from society for a long time. When I finally came up for air, I weighed over 300 lbs. My body and soul were damaged.

Obesity can be triggered by something like a childhood trauma, an injury, a genetic condition, a mental health condition, a metabolic issue, a socioeconomic issue and even by shame. Whatever triggers obesity, it impacts peoples’ lives and health. I hear people say that obesity is not a disease. Fat is just normal. Fat is not killing you. It is the internalized weight bias and shame that is killing you. Where does that shame come from? It comes from social stereotypes. It comes from the bias and stigma we experience on an ongoing basis. Yes, it can be part of it. But, the impact of obesity on my health is real. How can obesity be a social construction? Whether it is a disease, or a social construct matters to academics, but what matters to me is the ability to be here when my kids graduate and get married. What matters to me is my health.

We can debate whether obesity is a disease or not or whether calling obesity a disease will either reduce or increase weight bias and stigma, but it does not matter. These debates are delaying the ability for people with obesity to receive health care services. It can be a matter of life and death for individuals affected by obesity. Our lives are not academic projects. If you really think that obesity is not a disease and that our health is not affected by weight, that is your personal belief. I understand that there are people who identify as fat or as big persons. But do they have the right to question whether I have a disease or not? Even if you believe that it is the shame (weight bias and stigma) that is affecting my health, why do you deny me the right to seek support? Maybe it is the weight bias and shame that made me gain weight. Yes, there are studies that show that experiencing weight bias and stigma can increase obesity. But, so what? I still have to deal with the consequences of obesity because it is now affecting my health. Obesity is real. Obesity impacts my life. We do not need to argue about labels.

There are health professionals and fat acceptance advocates who do not accept that obesity is a disease. These debates seem to ignore that there is a person at the core of the discussion. Who is asking people with obesity what they think? At this point, it is fair to say that the voices of people with obesity are not invited in either the medical or the fat scholar debates. A core social value is to respect the rights of all human beings. Specifically, the Canadian Human Rights Act says:

… all individuals should have an opportunity equal with other individuals to make for themselves the lives that they are able and wish to have and to have their needs accommodated, consistent with their duties and obligations as members of society, without being hindered in or prevented from doing so by discriminatory practices based on race, national or ethnic origin, color, religion, age, sex, sexual orientation, gender identity or expression, marital status, family status, genetic characteristics, disability, or conviction for an offense for which a pardon has been granted…

Where do the rights of people with obesity fit in the Canadian Human Rights Act? Based on the understanding that obesity is a chronic disease, obesity could fit within the protected area of disability. But disability is also a stigmatized label.

Research shows that stigma is created when people distinguish and label human differences. These labels reflect dominant cultural beliefs and have a particular purpose. By placing people in distinct categories, we create degrees of separation between groups of people. It is an ‘us’ versus ‘them’ mentality. I am different than you and therefore you have the right to treat me differently. This idea that people with obesity are different or ‘*not normal*’ gives people the opportunity to treat us as ‘*abnormal*.’ This label has consequences for all of us living with obesity. People believe we did this to ourselves. We are not respected in society. We are seen as immoral persons because we have not taken care of our weight and we are somehow defective. We are not responsible persons and we should be punished for stepping outside of the ‘*normal boundaries*.’

Stigmatized persons experience status loss and discrimination that leads to various unequal health and social outcomes. Stigma has impacted my life chances. When I finally got help to address my depression, I went back to school to finish my university degree. I had trouble making friends because I could not participate in sports anymore. I did not have a group to belong to, so I was alone most of the time. When I entered the workforce, I went to many interviews and I could see the stares and negative attitudes among employers. I am certain I did not get many jobs because of my obesity. In my current employment, I have been passed for several promotions despite me having higher qualifications and better performance results. One co-worker complained to my manager that I smelled bad and requested an office move because he could not sit next to me. He did not tell me this to my face, but I overheard his comments in the washroom one day.

We must consider the power relations that underlie the ability of dominant groups to act on their biased attitudes and beliefs. We need weight bias and obesity stigma interventions to change institutional practices that work to disadvantage people with obesity in health care settings, workplaces, and schools.

### Laura: Shame and Vulnerability

Let’s unpack the shame that can trigger negative health behaviors. In my case, I hid in my room and ate until I weighed 250 lbs. The shame came from outside. People shamed me for my size since I was a baby. My parents put me on my first diet when I was about 12 months old because the doctor said I was too big for my age. They put me on a skim milk diet (as per the doctor’s advice). I ended up in the hospital. Just imagine what that did to my health. Science shows that yo–yo dieting is bad for your health. Well, I have been yo-to dieting since I was a baby.

The worry and shame that my parents felt about my size has been going on all my life. It made me feel unloved and alone. I have always been told that there is something wrong with my size. I responded to this shame by internalizing it. I believed my body was ugly, useless, worthless and abnormal. I disconnected from my physical body and began to hate it as if it was not part of me. But you cannot disconnect your body from you mind. As you start hating your body, you start hating yourself. You start hating everything about yourself. Not just your body. You hate who you are as a person. What do you think happens when you hate yourself that much? How do you reconcile this hatred in your mind? You simply try to survive. You try to repair the hate. But you do it by trying to change your body. By trying to look “*normal*.” By trying to fit into the “*normal BMI*” range. You try and try. You fail and fail. And when you fail, it is your fault.

What happens when you fail so many times is that you internalize the failure and start believing that you are just incapable of doing this. In my case, I developed alcoholism. That is how I coped with the shame. I was able to get help for alcoholism within the health care system because alcoholism is a disease, but I was not able to get help for obesity. I have been a recovered alcoholic for 25 years and I still have not been able to get help for my obesity.

Why is alcoholism a disease and not obesity? My alcoholism was also triggered by something else –the internalization of shame, the feelings of being out of control and that I was not “*normal*.” Doesn’t this sound familiar? It is the same shame that I have internalized that has led to me having obesity. But alcoholism is a disease. You don’t tell someone with alcoholism to deal with it alone. You provide support.

Once I realized that obesity is a chronic disease just like alcoholism, I asked my primary care doctor to refer me to the bariatric program. I was hopeful that I would have access to a team of health care professionals who are trained in obesity management and I finally would be able to get help. But that hope was shattered the moment I enrolled in the program. The bariatric program has basically continued to shame me. I expected these specialized health care professionals to be empathetic, knowledgeable, and supportive. Instead, they are arrogant, provide me with conflicting messaging and tell me that I just need to have bariatric surgery because that is the only treatment that will work for me. But, I don’t want to have surgery. So that means the program can’t help me. Where is the support?

From the moment, I walk into the bariatric clinic, the staff is rude to me. Nobody says hello. The dietitian implies that I am lying about my food intake because I have not lost weight. She doesn’t even look at my Fitbit or food journal. One dietitian told me that Fitbits are inaccurate so not to bother with it. But the first dietitian I met in the clinic told me to get one. Now this dietitian does not even want to look at it? I just spent $200 on this piece of equipment that she now claims is useless.

The psychologist and psychiatrist asked me if I think I need to talk to them. I said no because they just want to put me on antidepressants. Many psychiatric medications make you gain weight. I gained about 35 lbs while on medications. The nurse, on the other hand, tells me that if I don’t want surgery, the program can’t help me. How is this an obesity management program? We need to do better than this. People with obesity deserve better.

Like obesity, weight bias is always there, lingering. Self-stigma can come back anytime as a result of an experience of external obesity stigma. Unfortunately, obesity stigma can come from anyone, even from health professionals working in an institution that specializes in obesity. Although the goal is to eradicate weight bias all together, this may not be possible. There is always going to be a process of “us” and “them” at work in social interactions. However, taking examples from racism research, we know that racist ideologies have not changed completely but the manner in which racial prejudice is expressed has changed. It is not legal to discriminate against someone because of the color of their skin. This is where weight bias and obesity stigma interventions at the policy level are necessary. Legislations and policies to protect people with obesity from being discriminated against should be put in place.

## Master Narratives

Stories are selective, interpretative, and connective representations of human experience over time. They contribute to our self-identity and agency (i.e., our own understanding of who we are and what we do) (Linderman-Nelson, 2001). When we tell stories about our lives, we select elements in a way to represent a process of happening (beginning, middle, and end). We also interpret elements of a story by characterizing people, events, and places. The interpretation is always from a particular perspective or a way of seeing things. When we connect these elements of our stories over time, we create our self-identity.

Our identities, however, are developed through an interaction of how we see ourselves and how others conceive of us. How others conceive of us is influenced in part by master narratives – “*stories found lying about in our culture that serve as summaries of socially shared understandings*” (Linderman-Nelson, 2001). Many master narratives are morally benign and socially necessary. They help us make sense of ourselves and one another. There are, however, oppressive master narratives that can unfairly depict particular social groups (Linderman-Nelson, 2001). Oppressive narratives can create damaged social identities for groups and individuals, which can result in unjust treatment and deprivation of opportunity. This can in turn decrease life chances for individuals of a stigmatized group, resulting in health and social inequities. Importantly, when individuals internalize damaged identities through a process Linderman-Nielsen calls “infiltrated consciousness,” they can have implications on their own self-identity and agency (Linderman-Nelson, 2001). In the field of obesity, the concept of infiltrated consciousness had been described as internalized weight bias (i.e., holding negative beliefs about oneself because of one’s weight or size). Internalized weight bias has been found to have a distinct and direct effect on health outcomes, independent of any obesity-related health impairments (Pearl and Puhl, 2016). The link between experienced weight bias and internalized bias has important considerations for future interventions.

Through this cluster of individual stories, we can weave together a counterstory – a story that resists oppressive master narratives of people with obesity. Each story demonstrates how oppressive master narratives have been created and how individuals can challenge unjust assumptions that contribute to damaged social identities. The first task in constructing a counterstory for a group that faces stigmatization and oppression is to identify the oppressive master narratives that created damaged social identities. Based on the counterstories shared in this study, we found that the following master narratives may contribute to damaged social identities for people with obesity:

• Obesity is bad and by default people who have obesity are bad persons and a burden to society.
• People with obesity are “unhealthy” and “abnormal” because of their size.
• Obesity is a lifestyle choice.
• Body size or Body Mass Index reflects person’s health and/or health behaviors.

These oppressive master narratives are based on a lack of understanding of obesity as well unjust assumptions and stereotypes about individuals with obesity. The individual narratives in this study cast light on some unjust assumptions that create damaged social identities for individuals and groups affected by obesity.

• People with obesity cannot be healthy unless they achieve a “normal weight.”
• People with obesity do not exercise regularly and do not eat healthy.
• Individuals choose to be sedentary and to eat unhealthy foods – hence they choose to have obesity.
• Individuals can control their weight by eating healthy and exercising regularly.
• People with obesity lie about their eating and exercise habits.
• People with obesity are lazy, disgusting, ugly, smelly, and do not care about themselves.

These unjust assumptions about individuals with obesity can have significant consequences. The counterstories in this study reveal some of these consequences, including:

• Internalization of weight bias, where individuals with obesity come to believe in biased beliefs and unjust assumptions about obesity. The belief that their bodies are not “normal” and their desire to “fit in” and be “normal” leads to perpetual weight loss practices, as evident by the narratives in this study. Internalized weight bias and stigma can also lead to negative self-talk, feelings of shame and guilt that impacts their ability to engage in health promoting behaviors.
• External stigmatization via institutional and social practices can reduce individuals’ participation in education, employment, and in health promotion settings such as fitness and recreational centers.
• External stigmatization can also lead to unjust treatment by healthcare professionals, with serious consequences such as medical misdiagnosis.
• External stigmatization can take many forms, including verbal teasing and physical and mental abuse by family members, peers, health care professionals, work colleagues, and strangers.

Through their personal narratives, we observed that individuals find many ways to resist weight bias and stigma. Some strategies individuals use to resist weight bias and stigma include:

• Confronting their own internalized weight bias to find self-acceptance and self-respect. This gives individuals a sense of self-empowerment where they can redefine health in their own terms.
• Substituting master narratives of obesity as a lifestyle choice with chronic disease narratives where individuals can negotiate health versus weight. The substitution can be advantageous in many respects, including identifying factors that drive obesity that are beyond individual control, finding evidence-based disease management strategies that are unique to their individual needs, and seeking communities of support that they can use to renegotiate their self-identities.
• Resisting discrimination by identifying the power relations that underlie stigmatization and framing weight bias and stigma as a human rights issue.
• Resisting oppressive master narratives that depict people with obesity as engaged in unhealthy behaviors by inserting themselves in spaces where people with obesity are excluded (e.g., fitness and recreational centers).
• Resisting public bias, shaming and stereotyping by educating themselves and others about the complexity of obesity.
• Creating new communities of support to resist oppressive narratives and damaged identities and to educate themselves and others about the complexity of obesity.
• Contesting master narratives about obesity by opposing them with counterstories, both publicly and systematically. These counter stories are also effective in helping individuals with obesity to challenge their own self-perception, which has been affected by oppressive master narratives. This re-identification process permits people to repair their own damaged identities. It is important to note, however, that a counterstory can be used as a tool to repair a person’s internalized weight bias but sometimes it can be very difficult for someone to endorse a counterstory. It depends on the degree of internalization (Linderman-Nelson, 2001).

## Discussion

A successful counterstory can serve as an intervention to address damaged social identities for people living with obesity. There are several criteria for a successful counterstory. First, a good counterstory can pull apart master narratives that contribute to damaged social identities for people with obesity and replace them with credible, less morally degrading narratives. A counterstory must also be culturally digestible and widely circulated and taken up not only by those who are on the receiving end of stigma, but also by those who have benefited from it. Finally, a counterstory aims to free not only individuals but the entire group whose identity is damaged by an oppressive master narrative. Although a counterstory cannot end oppression, it can help re-identify a person or a group and in doing so freeing their agency.

Through this narrative inquiry, we intervened by focusing on the quality of lived experience, collaborated with participants to transform the narratives into counterstories and sought to lay the foundation for personal and social change. While conducting this research our lives continued to unfold and helped us see how we compose our lives within our familial, professional, and social situations. As we co-composed these stories with participants, they left an impact on our personal and professional lives. We have compared what we have been trained to “know” about obesity and what we have learned from living alongside individuals affected by obesity. We have questioned where our knowledge about obesity came from and how we adopted that knowledge. We have reflected on our role in contributing to weight bias and reflected on our own internalized weight bias. It has been a difficult journey, but we have developed more empathy and feel even more motivated to address oppressive obesity narratives.

In using interview and conversations recordings, memories, journals, field notes to compose the final research stories, we included actions and practices or things that we experienced together in the field. We composed the field texts over multiple interactions with each other and through reflections of earlier life experiences. Hence these final research products are the result of these research relationships and interactions. The final counterstories may therefore reflect multiple nested stories and reveal key aspects of weight bias and obesity stigma that were important to us as we negotiated the meaning of each story together. In one of the counterstories, the first author included herself in order to share how her own story has unfolded through this narrative inquiry process.

These counterstories offer a door to the personal, familial, professional, and social situations in which weight bias and stigma take place. In public health, we refer to this as context. Through this narrative inquiry, we have come to understand that context also includes personal relationships. Personal relationships impact what we know and what we do. As we lived alongside persons with obesity, we developed strong relationships with participants. These relationships have influenced what we know about weight bias, stigma and obesity and what we will do moving forward. We are now more sensitive to healthy lifestyle narratives, obesity labels and internalized weight bias and stigma, which are so pervasive in our culture. This experience has changed how we think and speak about obesity.

One of the key learnings of this research study for us was the transformative aspect of narrative inquiry. It is clear that both participants and researchers changed during the inquiry process. This research journey has made a difference in our lives, a key characteristic of narrative inquiry.

### Implications for Public Health

Using the three-dimensional space of narrative inquiry, we can position participants’ stories within place, time and social milieu, helping us to present the stories in a more pragmatic way that can inform future research and practice (Clandinin, 2013). Applying the three-dimensional space to these stories, we can see that individuals with obesity experience weight bias, stigma and discrimination across settings, including in their homes, schools, workplaces, recreational/fitness settings, and public health/health care settings. Weight bias experiences also take place across the lifespan and are influenced by institutional and social narratives (including public health narratives). These findings are consistent with existing weight bias and obesity stigma literature (Puhl and Heuer, 2009). Weight bias is deeply embedded in our culture leading to experiences of stigmatization, which cause disrespect, moral judgment, physical and mental abuse, social exclusion, and discrimination against people with obesity. Weight bias is used to enforce social norms and to try to get people to stay within normative boundaries. The normative boundaries about “*healthy weight*” or “*normal weight*” in our society can drive internalized weight stigma processes. For individuals who do not stay within the normative weight categories, this social label creates damaged social identities that causes experiences of discrimination, ultimately leading to health and social inequalities (Link and Hatzenbuehler, 2016).

Since weight bias is so ingrained in our culture, public health practice will inevitably be affected. As public health professionals, we need to critically reflect on these socio-cultural and professional biases and consider how they affect our practice. The implications of these counterstories for public health professionals depends on our own critical reflection skills and subjective realities. Below are a few implications for our personal public health practice that have emerged from this narrative inquiry.

• People with obesity experience weight bias across the lifespan and settings (home, school, work, health care settings, and communities), causing anxiety, low self-esteem, poor body image, social isolation, depression, suicidal acts and thoughts, medical illnesses and overall poor quality of life.
• Oppressive obesity narratives have become embedded in social institutions and systems perpetuating weight bias and stigma. Through our own professional practice, we can either reproduce weight bias and stigma or change systems to be more accepting and respectful.
• As public health professionals and researchers we have a responsibility to advocate and act to reduce health inequities for people living with obesity. But, we need theoretically driven and participatory interventions that can be implemented practically within current health and social systems.
• Working with individuals living with obesity to co-create counterstories aimed at changing damaged social identities can be transformative in terms of addressing internalized weight bias and creating empathy.
• Education about the multiple causes (social, cultural, psychological, and biological) of obesity needs to be incorporated into public and health domains in order to reduce weight bias in society.
• Conceptualizing obesity as a complex chronic disease requires comprehensive approaches that include prevention and management strategies. Reductionist approaches are not helpful and do not reflect the realities of people living with obesity. A focus to wellbeing of populations includes a need to support people with chronic diseases to live fulfilling lives.
• Weight bias and obesity stigma have direct and independent impacts on health and social outcomes for people with obesity. As such, weight bias and obesity stigma should be considered as key social determinants of health.

Through this narrative inquiry we learned that the fundamental driver of participants’ experiences with weight bias is a lack of understanding of obesity. This lack of understanding can be linked to public health narratives that oversimplify obesity as an unhealthy eating and lack of exercise issue. It also leads to social narratives that obesity is a self-inflicted choice and that it is up to individuals with obesity to address their own chronic disease. This lack of understanding can lead to people experiencing weight bias, stigma and discrimination. This narrative inquiry revealed people with obesity are treated differently by their families, friends, coworkers, health care providers, and even strangers. This lack of understanding of obesity has consequences for individuals’ conceptualization of their self-identity. Many participants internalized damaged social identities and felt abnormal. This also affected their self-confidence and self-worth. Weight bias internalization influenced participants’ emotional responses and triggered feelings of shame, blame, vulnerability, stress, depression, and even suicidal thoughts and acts. Participants responded to internalized weight bias by avoiding health promoting behaviors, hiding food, eating in secrecy, and isolating themselves from social and health promoting situations. Weight bias and stigma also hindered their obesity management process and rehabilitation and recovery strategies. Participants recovered from weight bias and stigma by developing self-compassion, self-acceptance and by engaging in efforts to resist damaged social identities and demand respect, dignity and fair treatment.

## Conclusion

Narrative inquiry is rooted in the epistemological assumption that knowledge is relational and that research relationships are built and negotiated. Thus, the understandings or meanings produced through this narrative inquiry are unique and never final. The meanings (findings) generated through this study will always be situated in the relations between the inquirers and the research participants. Our intent is not to make stories fit into a framework to make them easily disseminated as objective pieces of knowledge. We understand that the meanings (findings) from this study may extend beyond the inquiry process based on the position that readers are situated in when they experience the stories. Stories are never fully comprehensive or final because individuals experience stories differently based on their personal, social, and physical contexts. Our goal is not to make participants’ stories generalizable but rather to provide a deeper understanding of issues such as obesity, weight bias, stigma, and discrimination.

Based on the experiences of participants and researchers in this study, we conclude that narrative inquiry combined with the narrative repair model can be a transformative way to address internalized and experienced weight bias. However, future studies could also be implemented using quantitative internalized weight bias measures. This would allow us to quantitatively measure the changes in weight bias internalization before and after the narrative inquiry intervention.

## Ethics Statement

We obtained ethics approval from the University of Alberta Health Research Ethics Board. All participants provided informed consent after receiving a study information package prior to the conversations.

## Author Contributions

XRS conceptualized and designed the study, coordinated data collection, and drafted the initial manuscript. MF, TC, AS, and KR contributed to the design, data analysis, critical reflection, and contributed to writing and revising the manuscript. All authors approved the final manuscript as submitted and agreed to be accountable for all aspects of the work.

## Conflict of Interest Statement

AS has received compensation from Novo Nordisk and Bausch Health for service on advisory boards and has received compensation from Novo Nordisk and Merck for service on speakers’ bureaus, as well as travel reimbursement from both. The remaining authors declare that the research was conducted in the absence of any commercial or financial relationships that could be construed as a potential conflict of interest.

## References

[B1] Alberga A. S. Pickering B. J. Alix Hayden K. Ball G. D. C. Edwards A. Jelinski S. (2016a). Weight bias reduction in health professionals: a systematic review. *Clin. Obes.* 6 175–188. 10.1111/cob.12147 27166133

[B2] Alberga A. S. Russell-Mayhew S. von Ranson K. M. McLaren L. Ramos Salas X. Sharma A. M. (2016b). Future research in weight bias: what next? *Obesity* 24 1207–1209. 10.1002/oby.21480 27129601

[B3] Andreyeva T. Puhl R. M. Brownell K. D. (2008). Changes in perceived weight discrimination among Americans, 1995-1996 through 2004-2006. *Obesity* 16 1129–1134. 10.1038/oby.2008.35 18356847

[B4] Aston M. Price S. Kirk S. F. L. Penney T. (2012). More than meets the eye. Feminist poststructuralism as a lens towards understanding obesity. *J. Adv. Nurs.* 68 1187–1194. 10.1111/j.1365-2648.2011.05866.x 22070613

[B5] Bombak A. E. (2014). The contribution of applied social sciences to obesity stigma-related public health approaches. *J. Obes.* 2014:267286. 10.1155/2014/267286 24782921 PMC3982417

[B6] Browne N. (2012). Weight bias, stigmatization, and bullying of obese youth. *Bariatr. Nurs. Surg. Patient Care* 7:107 10.1089/bar.2012.9972

[B7] Clandinin D. J. (2013). *Engaging in Narrative Inquiry.* Walnut Creek, CA: Left Coast Press, Inc.

[B8] Clandinin D. J. Connelly M. (2000). *Narrative Inquiry: Experience and Story in Qualitative Research.* San Francisco, CA: Jossey-Bass.

[B9] Daníelsdóttir S. O’Brien K. S. Ciao A. (2010). Anti-fat prejudice reduction: a review of published studies. *Obes. Facts* 3 47–58. 10.1159/000277067 20215795 PMC6452150

[B10] Hatzenbuehler M. L. Phelan J. C. Link B. G. (2013). Stigma as a fundamental cause of population health inequalities. *Am. J. Public. Health* 103 813–821. 10.2105/AJPH.2012.301069 23488505 PMC3682466

[B11] Kirk S. F. L. Price S. L. Penney T. L. Rehman L. Lyons R. F. Piccinini-Vallis H. (2014). Blame, shame, and lack of support: a multilevel study on obesity management. *Q. Health Res.* 18 501. 24728109 10.1177/1049732314529667

[B12] Latner J. D. Barile J. P. Durso L. E. O’Brien K. S. (2014). Weight and health-related quality of life: the moderating role of weight discrimination and internalized weight bias. *Eat. Behav.* 15 586–590. 10.1016/j.eatbeh.2014.08.014 25215477

[B13] Lee M. Ata R. N. Brannick M. T. (2014). Review article: malleability of weight-biased attitudes and beliefs: a meta-analysis of weight bias reduction interventions. *Body Image* 11 251–259. 10.1016/j.bodyim.2014.03.003 24958660

[B14] Levin M. E. Potts S. Haeger J. Lillis J. (2018). Delivering acceptance and commitment therapy for weight self-stigma through guided self-help: results from an open pilot trial. *Cogn. Behav. Pract.* 25 87–104. 10.1016/j.cbpra.2017.02.002

[B15] Linderman-Nelson H. (2001). *Damaged Identities, Narrative Repair.* Ithaca, NY: Cornell University Press.

[B16] Link B. Hatzenbuehler M. L. (2016). Stigma as an unrecognized determinant of population health: research and policy implications. *J. Health Polit. Policy Law* 41 653–673. 10.1215/03616878-3620869 27127258

[B17] OC (2013). *RE: 5As of Obesity Management Framework and Resources.* Edmonton, AB: University of Alberta.

[B18] Pearl R. L. Hopkins C. H. Berkowitz R. I. Wadden T. A. (2018). Group cognitive-behavioral treatment for internalized weight stigma: a pilot study. *Eat. Weight Dis.* 23 357–362. 10.1007/s40519-016-0336-y 27787772

[B19] Pearl R. L. Puhl R. M. (2016). The distinct effects of internalizing weight bias: an experimental study. *Body Image* 17:38. 10.1016/j.bodyim.2016.02.002 26927688

[B20] Pearl R. L. White M. A. Grilo C. M. (2014). Weight bias internalization, depression, and self-reported health among overweight binge eating disorder patients. *Obesity* 22 E142–E148. 10.1002/oby.20617 24039219 PMC3954963

[B21] Pescosolido B. A. Martin J. K. Lang A. Olafsdottir S. (2008). Rethinking theoretical approaches to stigma: a Framework Integrating Normative Influences on Stigma (FINIS). *Soc. Sci. Med.* 67 431–440. 10.1016/j.socscimed.2008.03.018 18436358 PMC2587424

[B22] PHAC (2011). Curbing Childhood Obesity: A Federal, Provincial and Territorial Framework for Action to Promote Healthy Weights [Online]. Public Health Agency of Canada. Available at: http://www.phac-aspc.gc.ca/hp-ps/hl-mvs/framework-cadre/index-eng.php (accessed June 13, 2019).

[B23] Phelan J. C. Lucas J. W. Ridgeway C. L. Taylor C. J. (2014). Stigma, status, and population health. *Soc. Sci. Med.* 103 15–23. 10.1016/j.socscimed.2013.10.004 24507907 PMC4091623

[B24] Puhl R. Luedicke J. Peterson J. L. (2013). Public Reactions to Obesity-Related Health Campaigns: a randomized controlled trial. *Am. J. Prev. Med* 45 36–48. 10.1016/j.amepre.2013.02.010 23790987

[B25] Puhl R. M. Andreyeva T. Brownell K. D. (2008). Perceptions of weight discrimination: prevalence and comparison to race and gender discrimination in America. *Int. J. Obes.* 32 992–1000. 10.1038/ijo.2008.22 18317471

[B26] Puhl R. M. Heuer C. A. (2009). The Stigma of Obesity: a review and update. *Obesity* 17 941–964. 10.1038/oby.2008.636 19165161

[B27] Puhl R. M. Heuer C. A. (2010). Obesity stigma: important considerations for public health. *Am. J. Public Health* 100 1019–1028. 10.2105/AJPH.2009.159491 20075322 PMC2866597

[B28] Puhl R. M. Latner J. D. O’Brien K. Luedicke J. Danielsdottir S. Forhan M. (2015a). A multinational examination of weight bias: predictors of anti-fat attitudes across four countries. *Int. J. Obes.* 39 1166–1173. 10.1038/ijo.2015.32 25809827

[B29] Puhl R. M. Latner J. D. O’Brien K. S. Luedicke J. Danielsdottir S. Ramos Salas X. (2015b). Potential policies and laws to prohibit weight discrimination: public views from 4 countries. *Milbank Q.* 93 731–741. 10.1111/1468-0009.12162 26626983 PMC4678937

[B30] Ramos Salas X. Alberga A. Cameron E. Estey L. Forhan M. Kirk S. F. L. (2017a). Addressing weight bias and discrimination: moving beyond raising awareness to creating change. *Obes. Rev.* 11 1323–1335. 10.1111/obr.12592 28994243

[B31] Ramos Salas X. Forhan M. Caulfield T. Sharma A. M. Raine K. (2017b). A critical analysis of obesity prevention policies and strategies. *Can. J. Public Health* 108 e598–e608. 10.17269/cjph.108.6044 31823280 PMC6972457

[B32] Sutin A. R. Stephan Y. Terracciano A. (2015). Weight discrimination and risk of mortality. *Psychol. Sci.* 26 1803–1811. 10.1177/0956797615601103 26420442 PMC4636946

[B33] Teachman B. A. Brownell K. D. (2001). Implicit anti-fat bias among health professionals: is anyone immune? *Int. J. Obes. Relat. Metab. Dis.* 25:1525. 10.1038/sj.ijo.0801745 11673776

[B34] Teachman B. A. Gapinski K. D. Brownell K. D. Rawlins M. Jeyaram S. (2003). Demonstrations of implicit anti-fat bias: the impact of providing causal information and evoking empathy. *Health psychol.* 22 68–78. 10.1037//0278-6133.22.1.68 12558204

[B35] Thompson L. Kumar A. (2011). Responses to health promotion campaigns: resistance, denial and othering. *Crit. Public Health* 21 105–117. 10.1080/09581591003797129

[B36] WHO (2000). *“Obesity: Preventing and Managing the Global Epidemic,”* in *World Health Organization.* Technical Report Series 894. Geneva: World Health Organization. 11234459

